# A Precise Closed-Loop Controlled ZnO Nanowire Resonator Operating at Room Temperature

**DOI:** 10.3390/mi13060952

**Published:** 2022-06-16

**Authors:** Xianfa Cai, Lizhong Xu

**Affiliations:** School of Mechanical Engineering, Yanshan University, Qinhuangdao 066000, China; caixianfa@stumail.ysu.edu.cn

**Keywords:** nanoresonator, electromagnetic excitation, closed-loop control, quality factor, phase noise, equivalent circuit model

## Abstract

To realize the real-time measurement of masses of nanoparticles, virus molecules, organic macromolecules, and gas molecules, and to analyze their physical and chemical properties, a ZnO nanowire (NW) resonator operating at room temperature with an ultrahigh resonant frequency, real-time detection, and high precision was designed and developed in this study. The machining method is simple and easy to integrate into an integrated circuit. A closed-loop detection system based on a phase-locked loop (PLL) and frequency modulation technology (FM) was used to perform closed-loop testing of electromagnetically excited ZnO NW. The first-order resonance frequency of the resonator was 10.358 MHz, the quality factor *Q* value was about 600, the frequency fluctuation value *f*_RMS_ was about 300 Hz, and the FM range could reach 200 kHz. The equivalent circuit model of the resonator was established, the parasitic parameters during the test were obtained, and the frequency accuracy and phase noise of the resonator were analyzed and tested. The experimental results show that the closed-loop system can automatically control the resonator in a wide range of frequency bands, with good tracking performance of the resonant frequency, small frequency fluctuation, and low phase noise level.

## 1. Introduction

Recent advances in micro- and nanomanufacturing have enabled the miniaturization of semiconductor-based mechanical resonators, which facilitates their on-chip integration with electronic components [1,2]. However, to be fully compatible with very large-scale integrated circuits (VLSI), the resonant elements should eventually be further reduced in size to the range of nanoelectromechanical systems (NEMSs) for direct compatibility with individual transistors in size. NW resonators have many advantages, such as high sensitivity, low energy consumption, and fast response speed [3,4], which is an important development direction of resonators. Its strong sensitivity can reach the level of measuring several or even a single inert gas atom [5,6,7], and its sensitivity can be increased to 1012 times higher than that of a traditional quartz crystal monitor [6]. It can be widely used in ultrahigh sensitivity quality detection [7], force detection [8], acceleration detection [9], high-frequency signal generation and processing [10], and high-speed logic operation [11]. It is clear that NW resonators provide unprecedented abilities to measure individual neutral molecules or atoms and will find many interesting applications in mass spectrometry and atomic physics, with great significance for environmental monitoring, industrial production, medical diagnosis, and defense and military fields.

Noise suppression is critical to closed-loop oscillators, which are the core of many current technologies such as self-sustaining vibration and sensing [12]. Closed-loop detection technology is of great significance for real-time measurement, improving the intelligence and measurement accuracy of sensors [13]. Noise suppression and closed-loop real-time measurements of nanomechanical oscillators have proven to be very challenging, mainly because the electrical signal is weak when reducing the size of the NEMS components, which makes it extremely difficult to use and control the signals generated by NEMS motion in the face of inevitable stray reactance [14]. A large number of nanoresonators have problems with low real-time performance and low accuracy. In order to solve this problem, some studies were performed in which the influence of the external driving force on the resonance characteristics such as the natural frequency and quality factor of the resonator was investigated [15], and the measurement circuit was improved to reduce the frequency fluctuation and improve the measurement accuracy [16,17]. Several studies have been carried out in the literature [13,18,19] on closed-loop control of the resonator, in which researchers studied the dynamics of the adsorption mass on the upper surface of the resonator, analyzed the adsorption noise through closed-loop measurement, and observed the diffusion noise of the adsorption mass. However, there are some problems such as low *Q* value of the resonator, low precision of closed-loop control, only operation in ultralow temperature environment, small range of trackable frequency, and difficulty in integrating the manufacturing process of the resonator into integrated circuits.

Therefore, in this paper, a ZnO NW resonator was fabricated by top–down machining. This processing method has less damage to the NWs, so the dissipation of the resonator is small, and the ZnO NW resonator can operate at room temperature with small frequency fluctuation, which is conducive to the formation of closed loops. A closed-loop detection system based on a phase-locked loop and frequency modulation technology was used to test the electromagnetic excited ZnO NW. An equivalent circuit model was established to analyze the quality factor, frequency accuracy, and phase noise of the resonator. Experimental results show that the proposed resonator has low phase noise, high closed-loop stability, and high measurement accuracy. The improvement of NW resonators processing method and the design of high precision and large frequency range closed-loop test system opens new opportunities for NW resonators integration in chips [20], real-time measurement of particle mass [4], and real-time monitoring of organic or biomolecular chemical reactions [21].

## 2. Microfabrication and Closed-Loop System Design

### 2.1. Fabrication of ZnO NW Resonators

**Figure 1:**
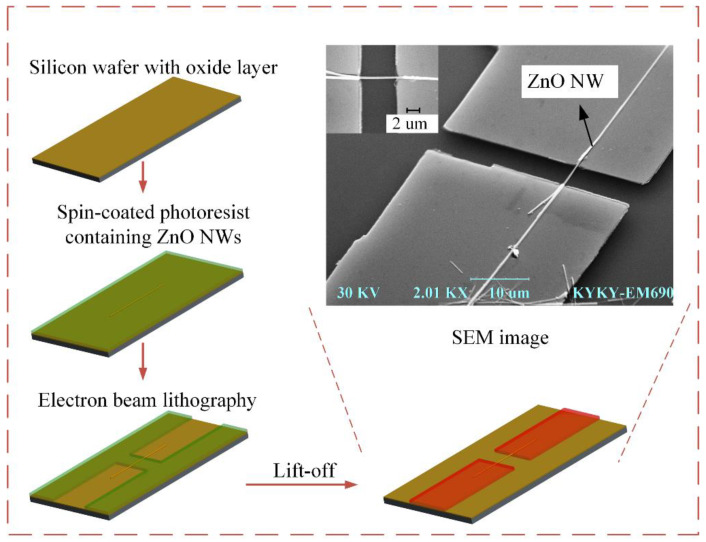
Fabrication process of ZnO NW resonator.

ZnO NW is a one-dimensional semiconductor material with excellent chemical, mechanical, and thermal stability [22]. The ZnO NWs used were confirmed to be single crystal, with no twin or defect by transmission electron microscopy. These NWs belong to semiconductors and have mature growth and manufacturing technology [23]. In this study, nanoelectromechanical processing techniques such as electron beam exposure technology and lift-off process [24,25,26] were used to fabricate ZnO NW resonators clamped at both ends by a top–down processing method. This process avoids the potential damage of NWs due to surface tension in wet etching. The production process is shown in Figure 1. First, the silicon wafer was activated with a uniform surface oxidation layer of 2 μm silicon oxide as the substrate, and the substrate was placed in a 460 K environment for 20 min, so as to ensure good electrical insulation, but also the effective transfer of electrons to the earth during scanning electron microscopy. Then, the substrate was coated with a layer of PMMA photoresist with a thickness of about 400 nm and pre-baked (temperature 394 K, time 20 min). Then, the ZnO NWs were dispersed on a PMMA photoresist with a diameter of 80~120 nm, a length of 7–30 μm, and an aspect ratio of 60~300. The ZnO NWs were then coated with a PMMA photoresist of approximately 400 nm thickness (the thickness of the adhesive was sufficient for the subsequent lift-off process). After pre-drying, electron beam graphic exposure was carried out on both ends of the NWs in a scanning electron microscope. After development and fixing, the hard film was processed (temperature 364 K, time 20 min), which was helpful to improve the strength of the mold. Then, 400 nm to 800 nm gold was evaporated to provide clamping and lead function for the NWs, and the evaporation rate was as slow as 0.4 Å/s to improve the tightness of the material inside the electrode. Then, the PMMA photoresist was dissolved by acetone using a lift-off process to obtain the NW resonator, with two ends clipped together. Finally, the surface of the electrode was coated with negative adhesive and graphically exposed by an electron beam, so that a protective film was formed on the surface of the electrode to reduce the oxidation of the electrode and reduce the noise caused by the charge interference of air molecules.

### 2.2. Principle of Closed-Loop Measurement and Control System

To realize the real-time closed-loop measurement of resonant frequency, a closed-loop control strategy combining PLL and FM is used. The approximate resonant frequency of the resonant beam is [27]

(1) $$f=0.9395\frac{d}{L^{2}}\sqrt{\frac{E}{\rho }}$$

where *f* is the first-order natural frequency of the nanobeam; *d* is the diameter of the ZnO NW; *L* is the length of the resonant part of the NW; *E* is the elastic modulus of the ZnO NW, usually 2.1 × 10^11^ Pa [27]; *ρ* is the density of ZnO NW, usually 5.67 × 10^3^ kg/m^3^ [27].

**Figure 2:**
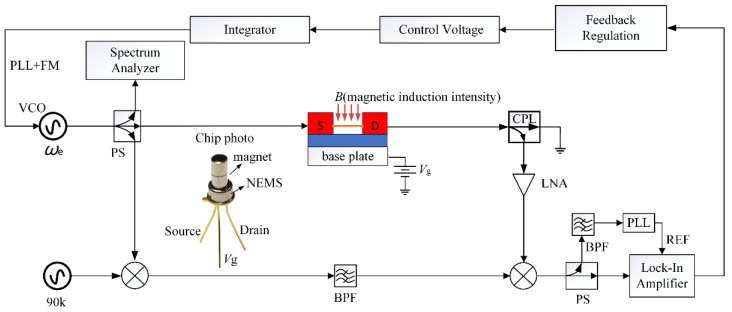
Closed-loop measurement and control system for ZnO NW resonator. VCO: signal generator (HP8648C, maximum FM voltage is ±1 V), PS: power divider, BPF: bandpass filter, LNA: low noise amplifier, ⊗: mixer. *V*_g_: substrate voltage, used to apply a biasing force to the nanobeam. Directional coupler (CPL) selects ZEDC-15-2B; the coupling degree is 15 dB.

As shown in Figure 2, a voltage-controlled oscillator (VCO) generates a sinusoidal voltage signal *ω*_e_ with a frequency equal to the calculated resonant frequency. A uniform and constant magnetic field is applied to the transverse vibration direction of the NW to drive the NW to vibrate, and the information generated by the NW vibration can be extracted by detecting the change in the induced electromotive force [28]. In the high-frequency circuit, the input and output impedances and connecting lines of the modules are all matched with 50 Ω impedance. In the detection circuit, a directional coupler needs to be added to detect the induced electromotive force without forming loop interference, and the directional coupler also plays the role of impedance transformation. The VCO output signal can be mixed with the reference signal Δ*ω* to obtain the voltage signal of *ω*_e_ ± Δ*ω* through the bandpass filter, which is mixed with the voltage signal of the resonator output, can obtain the low-frequency signal containing the frequency close to Δ*ω*. After passing the low-frequency signal through a bandpass filter, it is fed into a PLL, which can automatically track to *f*_LOCK_, the frequency component with the maximum amplitude of the signal. The PLL lock frequency *f*_LOCK_ and Δ*ω* values are compared by a lock-in amplifier, and the value *f*_LOCK_ deviating from Δ*ω* is equal to the fluctuation of the resonant frequency. The lock-in amplifier can output a corresponding adjustment voltage according to the difference, and the feedback adjustment module will operate the adjustment voltage through a preset program to obtain the Mod voltage for frequency modulation of the signal source. The Mod voltage is then sent through an integrator to the VCO, which adjusts the excitation signal’s frequency *ω*_e_ in real time to match the resonant frequency of the NWs. In this dynamic FM process, the frequency of the excitation signal output by VCO is not fixed. PLL, combined with FM, can continuously and rapidly modify the value of the excitation signal until the frequency of the excitation signal is the same as the resonant frequency of the NW, and *f*_LOCK_ can steadily and rapidly approach Δ*ω*. When the *f*_LOCK_ detected in the lock-in amplifier shakes slightly around, Δ*ω*, the entire system is considered to be in a locked state, and the frequency output by the VCO is the resonant frequency of the NW. When the resonant frequency of the NW changes, the system can quickly detect the value of the excitation signal frequency deviating from the resonant frequency of the NW and adjust the frequency so that the excitation signal can track the resonant frequency of the NW in time.

In the lock-in amplifier, the cross-correlation operation between the signal to be measured and the reference signal is realized by electronic components such as an integrator, so as to obtain the amplitude of the signal at the same frequency as the reference signal in each frequency component of the signal to be measured. Affected by the settings of measurement parameters such as the time constant of the lock-in amplifier, the measurement system of the lock-in amplifier responds differently. To make the *Q* value measurement more accurate and ensure the stability of the waveform, the time constant was set to 100 ms. Generally, when the low-frequency signal is used, the self-adjustment range of automatically detected resonant frequency is not very large, and the FM range can be set very small. Since the detection frequency range of the lock-in amplifier used in this paper was 1 mHz~102 kHz, and the fluctuation value obtained through experiments was less than 10 kHz, the frequency of the reference signal Δ*ω* could theoretically be set to 1 mHz~92 kHz. However, in order to take the realization of the function of the closed-loop strategy circuit into account, FM was set to 1 kHz (Mod voltage 1 V linearly corresponds to FM 1 kHz), and Δ*ω* was set to 9 kHz. Assuming that *f*_LOCK_ detected by the lock-in amplifier was 8.9 kHz, the frequency *ω*_e_ of the excitation signal of the VCO should be reduced by 0.1 kHz, and the FM voltage should be −0.1 V. Likewise, with *f*_LOCK_ at 9.1 kHz, the Mod value should be 0.1 V. Therefore,

(2) $$\mathrm{Mod}=\left(0.001\times f_{\mathrm{LOCK}}-9\right)\left(V\right)$$

The resonant frequency changes greatly at high frequencies, so the FM range of the VCO was set to 200 kHz. Considering the bandwidth of the device and the implementation of the algorithm for the subsequent frequency modulation voltage Mod, Δ*ω* was set to 90 kHz when the resonant frequency was above 1 MHz. Mod value should be

(3) $$\mathrm{Mod}=\left(5\times 10^{-6}\times f_{\mathrm{LOCK}}-0.45\right)\left(V\right)$$

As the closed-loop circuit has a strong self-adjustment ability, the circuit in the frequency modulation link does not need high precision. The whole closed-loop feedback circuit is equivalent to a closed-loop system controlled according to the frequency difference, which is composed of three basic circuits: frequency discriminator, loop filter, and VCO. The closed-loop feedback regulation system has two regulation processes—one is the tracking process and the other is the capturing process. The former occurs when the loop is locked, during which, if the natural frequency of the NW resonator changes, the excitation signal can track the natural frequency of the NW resonator in time; the latter is the process in which the loop goes out of and back into the lock when the frequency of the excitation signal deviates from the natural frequency of the NW resonator. The closed-loop feedback system can track the natural frequency of the NW resonator. When the loop is locked, the frequency of the excitation signal output by the VCO is equal to the natural frequency of the NW resonator, that is, the steady-state frequency difference is zero. In this study, the lock-in amplifier plays the role of frequency detection in the entire closed-loop loop. It detects the fluctuation of the resonant frequency and outputs the DC regulation voltage. The DC regulation voltage is passed through a feedback controller to provide the Mod voltage (Equations (2) and (3)). This closed-loop feedback system has the ability to detect and feedback the weak signal generated by the NW resonator because it adopts a lock-in amplifier, which is difficult for other automatic control systems to achieve.

## 3. Measurement and Discussion

### 3.1. Closed-Loop Experiment Process

**Figure 3:**
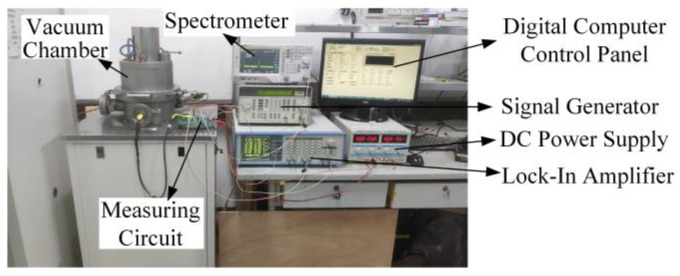
Photo of closed-loop test equipment.

One of the fabricated resonators was measured via SEM, and *L* was 6.1 μm, *d* was 70 nm, and the resonant frequency of the resonator was calculated to be 10.76 MHz by Formula (1). To verify the closed-loop measurement and control system, the resonator was connected to the closed-loop system with wire bonding. As shown in Figure 3, the measurement was carried out in a closed container in a near-vacuum condition (1 × 10^−3^ Pa) at room temperature. In order to reduce the impact of temperature fluctuations on the resonant frequency, water was circulated on the surface of the vacuum cavity, and the resonator in contact with the vacuum cavity was insulated via heat transfer. Through testing, the surface temperature of the resonator was maintained at 21.0~21.2 °C. The resonator should be placed in a near-vacuum airtight container for more than 12 h to minimize some impurities adsorbed on the NW, thereby reducing frequency fluctuations and improving the quality factor of the resonator.

Before closed-loop measurement, an open-loop test was performed on the resonator to determine its resonant frequency more accurately. During the measurement, the FM function of VCO was turned off, and a sweep driving voltage with a voltage amplitude of 5 V was set (the voltage needed to be greater than 1 V for the NW resonators to generate enough strong signals for the closed-loop system), the magnetic field intensity *B* was set to 0.5 T, and *V*_g_ was set to 2 V (*V*_g_ was just the DC bias of the driving signal and was used to induce an initial deformation of the resonant beam). The resonant frequency of the resonator was determined by observing the voltage peak of the signal received by the lock-in amplifier. Through open-loop measurement, the first-order resonant frequency of the resonator was 10.352 MHz, the *Q* value was about 600, and the measurement results were in good agreement with the calculated data, indicating that the measurement was carried out in the bending vibration of the NW. The FM function of the VCO was turned on, and through the closed-loop test, the resonant frequency of the resonator was found to be 10.358 MHz. It was revealed that the resonant frequencies detected by using the open-loop and the closed-loop tests were not the same, which may be because the open-loop detection of the resonant frequency is affected by the parasitic capacitance and resistance of the test system, while the closed-loop detection can well avoid the influence of these factors on the test.

### 3.2. Measurement of Resonant Frequency Fluctuations

**Figure 4:**
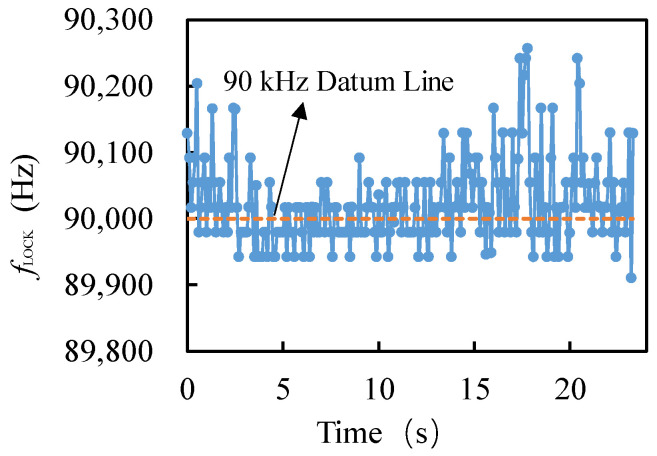
Variation of *f*_LOCK_ over time.

The closed-loop test is different from the open-loop test using a network analyzer, which directly detects the change in the capacitance value between the NW and the substrate caused by the vibration of the NW, so as to directly detect the resonant frequency of the resonator, and can automatically track this resonant frequency. By recording the *f*_LOCK_ of the lock-in amplifier locking signal, the frequency fluctuation of the resonator and the tracking of the resonant frequency via the closed-loop detection system can also be obtained. As shown in Figure 4, it can be seen that, due to the self-adjustment of the system, the locking frequency *f*_LOCK_ continuously fluctuated around 90 kHz with time, and the fluctuation value *f*_RMS_ was about 300 Hz, which is basically consistent with the oscilloscope detection result and close to some frequency fluctuations values detected in ultralow temperature environments [14,15,16]. It was proved that the resonant frequency of the NW resonator is stable and the closed-loop system can track its resonant frequency well. 

### 3.3. Measurement of Resonator Phase Noise

Thermomechanical noise originates from random thermal actuation in NEMS, causing short-term instability in frequency and ultimately manifesting in the phase noise of the resonator. Although limited by thermomechanical fluctuations at room temperature (the ultimate performance of the NEMS oscillator is limited by the thermomechanical fluctuations of the device), it is possible to achieve the inherent phase noise limit of the NEMS by using advanced resonator design methods to reduce noise and increase the *Q* value [29].

Although the surface temperature fluctuation of the resonator can be controlled at 0.2 °C during the experiment, and the machining accuracy can be improved as much as possible to reduce the asymmetry of the resonator structure, the temperature fluctuation still affects the frequency stability of the resonator. To measure this effect, the effect of temperature change on the resonant frequency was recorded during the experiment: The water circulation system was turned off, and the change in resonant frequency with temperature was recorded. The material characteristics of the NWs themselves, noise (driving signal noise and phase noise), and thermomechanical noise (which is smaller than the first two items) brought by using the measurement devices all bring fluctuations to the resonant frequency, and the fluctuations are affected by temperature [30,31]. When the average value of resonant frequency was recorded with the change in temperature, it was found that |d*f*/dT| ≤ 1 kHz/°C, that is, when the temperature fluctuation of the resonator surface can be controlled within 0.2 °C, the influence of temperature on the resonant frequency was less than 200 Hz. This value is less than the *f*_RMS_ caused by the driving signal noise and phase noise, indicating that the material characteristics of the resonator itself and the noise (driving signal noise and phase noise) caused by the measuring device have leading roles in the frequency fluctuation.

**Figure 5:**
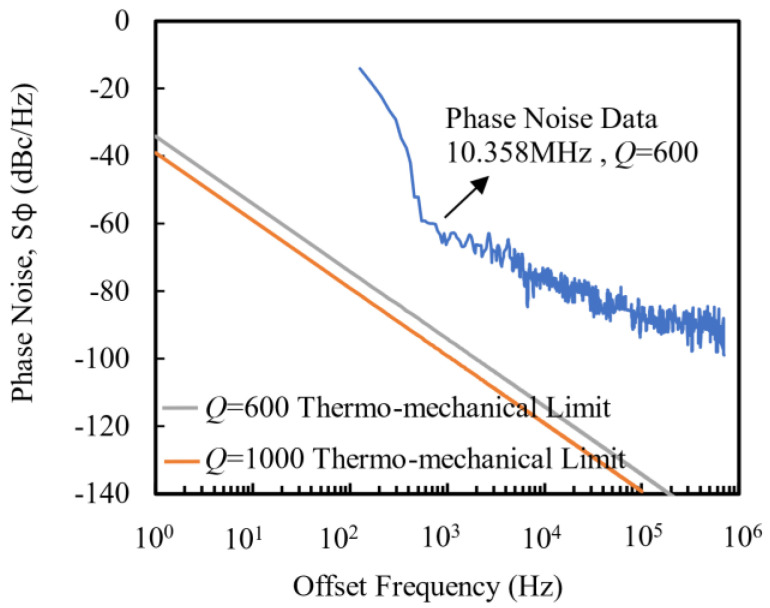
Phase noise spectrum of resonator for closed-loop test.

The phase noise of the resonator was measured. As can be seen from Figure 5, when the frequency deviated from the center frequency within 1 kHz, the phase noise decreased rapidly with the increase in the deviation frequency Δ*f*, and the proportional relationship between the phase noise and the frequency was greater than 1/*f* ^3^. When the Δ*f* was greater than 1 kHz, the phase noise was approximately proportional to 1/*f*. When Δ*f* was greater than 100 kHz, the phase noise was limited by thermomechanical fluctuations at room temperature, and the measured phase noise was close to the bottom line of the phase noise (the limits of thermomechanical phase noise at *Q* = 600 and 1000 are given in Figure 5 [32]). This indicates that the phase noise performance of the NW resonator, which has not been optimized yet, reached the advanced level of the NEMS resonator with fixed ends [32].

### 3.4. Obtaining the Parasitic Parameters of the Resonator

#### 3.4.1. Establishment of Equivalent Circuit Model

**Figure 6:**
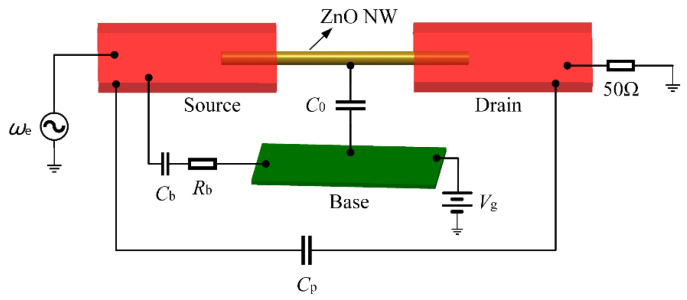
Schematic diagram of the parasitic parameters introduced by the test.

The performance evaluation indicators of resonators include resonant frequency, *Q* value, frequency fluctuation, etc. [30,31]. Many studies in the literature have analyzed the influence of the internal mechanism (the clamping condition at the end of the resonant beam, the natural characteristics and surface condition of the beam), and the external mechanism of the resonant beam (the air pressure and temperature in the experiment) on the *Q* value [30,31,32]. The *Q* value of the resonator can be improved by increasing the thickness of the clamping end, increasing the length–diameter ratio of the resonant beam, reducing surface impurities, and operating in an ultralow temperature environment. The parasitic parameters introduced in the process of device manufacturing and testing can lead to serious distortion of the device test results. Many measurement methods, such as an electronic reference circuit for signal subtraction [33,34] and regulating electrostatic stiffness [35], were proposed for reducing the influence of parasitic parameters on resonators. In this study, we mainly analyzed the influence of parasitic parameters on the resonator *Q* value. As the bias voltage and AC excitation were loaded at the same electrode, the resonator could be equivalent to a single-port equivalent circuit. Considering the parameters of the resonator itself, the parasitic parameters generated during the test of the resonator are shown in Figure 6. *C*_0_ represents the overlapped static capacitance between the NW and the substrate. *C*_b_ and *R*_b_, respectively, correspond to the coupling capacitance and the corresponding parasitic resistance of the substrate when the signal lead-out line of the resonator and the base electrode passes through the substrate. *C*_p_ represents the parallel parasitic capacitance (including the parasitic capacitance of the substrate and the solder joint capacitance, etc.) generated by the device during the test [36].

**Figure 7:**
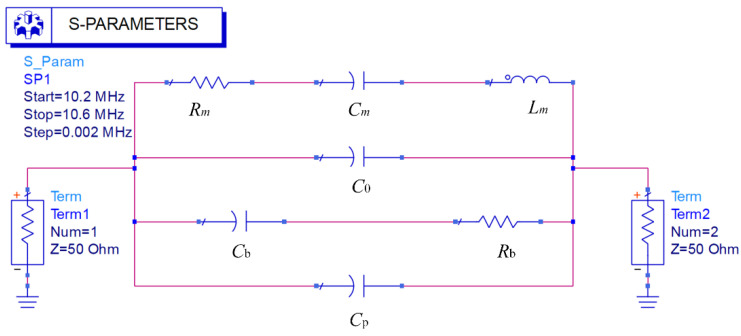
Equivalent circuit model of the resonator.

In order to obtain the value of parasitic parameters and explore its influence on the *Q* value, according to the method of electromechanical analogy, the equivalent circuit model of the resonator was obtained by comparing the mechanical parameters of each of the components of the resonator with electrical parameters. To facilitate further simulation, the equivalent circuit model was established by using advanced design system (ADS) software. As shown in Figure 7, *R_m_* is the equivalent resistance, corresponding to the damping coefficient of the resonator. The equivalent capacitance *C_m_* corresponds to the reciprocal of the equivalent stiffness of the resonant beam. The equivalent inductance *L_m_* corresponds to the equivalent mass *m* of the resonant beam.

#### 3.4.2. Calculation of Equivalent Circuit Model Parameters

To analyze and simulate the whole test system, it is necessary to determine the electrical parameters such as resistance, capacitance, and inductance according to the resonator parameters shown in Table 1.

The relationship between electrical parameters such as resistance, capacitance, and inductance and resonator parameters in the equivalent circuit model is as follows [37]:

(4) $$\begin{array}{c} R_{m}=\frac{\sqrt{km}}{Q\eta^{2}}\\ k={\left(2\pi f\right)}^{2}m\\ m={\int_{0}^{L}\rho A\left[\phi \left(x\right)\right]}^{2}dx\approx 0.5\rho AL \end{array}\}$$

where *A* is the transverse cross-sectional area of the NW; *ϕ*(*x*) is the first-order modal function of the NWs. *η* is the electromechanical coupling coefficient, which can be expressed as

(5) $$\eta =\frac{U_{0}\varepsilon_{0}A_{0}}{D^{2}}$$

where *ε*_0_ is the dielectric constant of vacuum, usually set as *ε*_0_ = 8.85 × 10^−12^ F/m; *A*_0_ is the effective area of the base directly opposite the NW. Equivalent capacitance and equivalent inductance can be expressed as

(6) $$\begin{array}{l} C_{m}=\frac{\eta^{2}}{k}\\ L_{m}=\frac{m}{\eta^{2}} \end{array}\}$$

The overlapping static capacitance between the NW and the substrate can be expressed as

(7) $$C_{0}=\frac{\varepsilon_{0}A_{0}}{4\pi k_{0}D}$$

where *k*_0_ is the static force constant, *k*_0_ is often taken as 8.99 × 10^9^ N. The solution results of equivalent circuit model parameters are shown in Table 2.

#### 3.4.3. Identification of Parasitic Parameters of Resonator

The equivalent circuit model shown in Figure 7 was used to identify the parameters of *C*_b_, *R*_b_, and *C*_p_. The identification method adopts the least square method, that is, to obtain

(8) $$\min\sum_{i=1}^{N}{\left[{\left(Zrdata(i)-Zr(i)\right)}^{2}+Zidata(i)-Zi(i)\right)}^{2}]$$

where *Zrdata* and *Zidata*, respectively, represent the real and imaginary parts of measured impedance values, *Zr* and *Zi*, respectively, represent the variables to be identified in the real and imaginary parts of impedance, and *N* represents the number of samples.

In a near-vacuum (1 × 10^−3^ Pa) environment and with a frequency band in the range of 1 MHz~25 MHz, the alternating voltage was applied to the resonator, and the lock-in amplifier was used to measure the output current of the resonator to calculate the impedance value of the resonator.

Impedance value *Zi* of each branch in the equivalent circuit model and total impedance *Z* are:

(9) $$\begin{array}{c} Z_{1}=R_{m}+j\omega L_{m}+\frac{1}{j\omega C_{m}}\\ Z_{2}=\frac{1}{j\omega C_{0}}\\ Z_{3}=\frac{1}{j\omega C_{b}}+R_{b}\\ \begin{array}{c} Z_{4}=\frac{1}{j\omega C_{p}}\\ Z=\frac{Z_{1}Z_{2}Z_{3}Z_{4}}{Z_{1}Z_{2}Z_{3}+Z_{1}Z_{2}Z_{4}+Z_{1}Z_{3}Z_{4}+Z_{2}Z_{3}Z_{4}} \end{array} \end{array}\}$$

**Figure 8:**
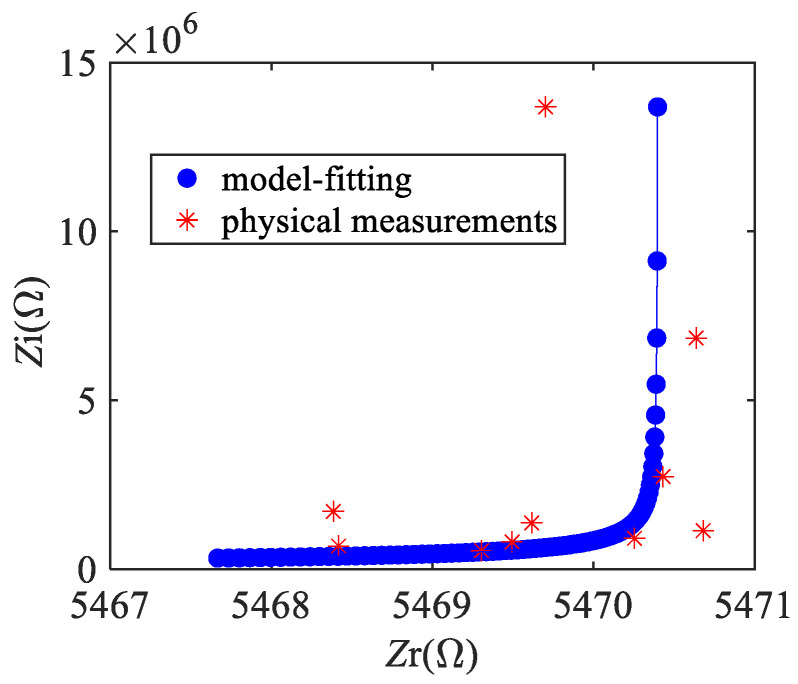
Cole–Cole diagram of equivalent circuit model in frequency band 1~25 MHz.

Using MATLAB software to identify the parasitic parameters by the least square method, *C*_b_ was obtained as 32 fF, *R*_b_ was 2.85 kΩ, and *C*_p_ was 41 fF. Based on the Cole–Cole diagram of the equivalent circuit model at the 1~30 MHz frequency band, as shown in Figure 8, it can be inferred that the impedance characteristics of the resonator model obtained through parameter identification fit well with the values obtained by experimental measurement.

#### 3.4.4. Influence of Parasitic Parameters on *Q* Value

**Figure 9:**
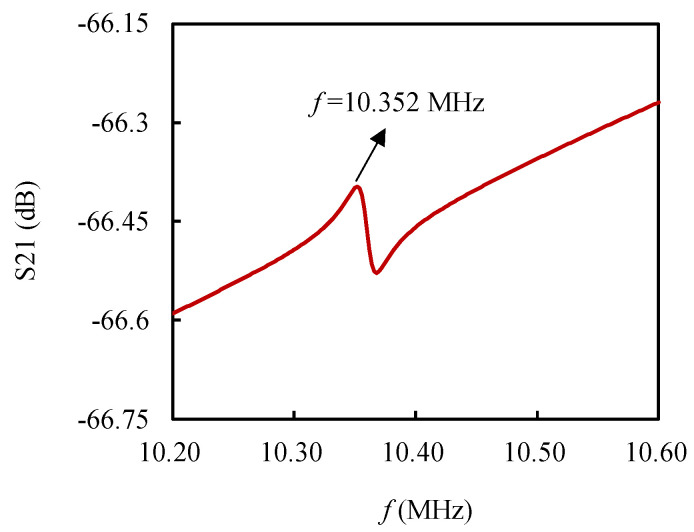
Simulation results of the equivalent circuit model.

The equivalent circuit model of this resonator was simulated with ADS software. As shown in Figure 9, the relationship between the transmission coefficient (S21 parameter) of the resonator and the excitation frequency *f* was obtained after the S-parameter simulation. Due to the parasitic resonance caused by the presence of *C*_0_ and *C*_p_, there are two resonance peaks in Figure 9, of which the parallel resonance peak of the resonator is on the right. According to ADS simulations and related references, the parallel resonance peak is caused by parasitic parameters in open-loop detection, and there are generally two resonance peaks. The natural frequency of the resonator obtained by simulating the model with ADS software was 10.352 MHz, and the *Q* value was about 510, which is not much different from the experimentally measured value. It should be noted that the simulation results were closer to the open-loop test results, which may be due to the fact that the open-loop test is more susceptible to the influence of the peripheral circuits during measurement than the closed-loop test.

**Figure 10:**
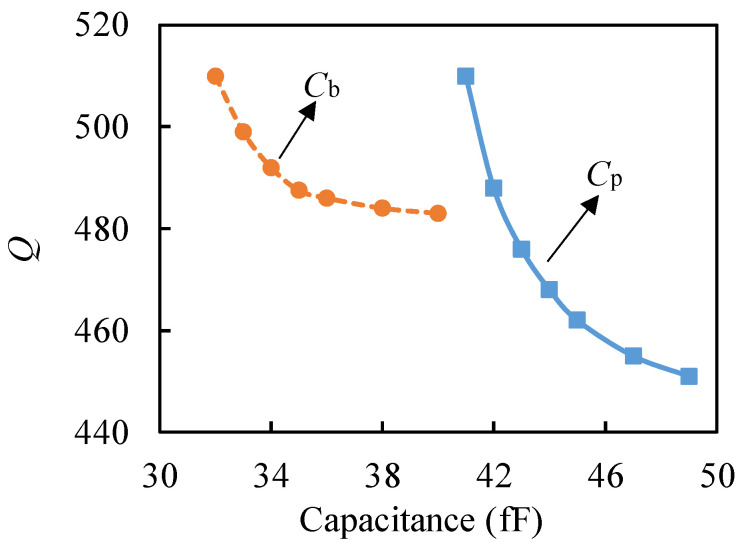
Effects of *C*_b_ and *C*_p_ on *Q*.

ADS simulation shows that, as shown in Figure 10, when *C*_b_ and *C*_p_ values increased by 10%, the resonator *Q* value decreased by 4.7% and 9.4%, respectively, and the influence of *C*_b_ and *C*_p_ on *Q* value decreased with the increase in *C*_b_ and *C*_p_ values. It shows that the increase in *C*_b_ and *C*_p_ reduced the resonator *Q* value, and the influence of *C*_p_ on the *Q* value was nearly twice that of *C*_b_. Based on the results of this study, suppression of parasitic resonance can be achieved by reducing the static capacitance *C*_0_ and parasitic capacitance *C*_p_, or by using the closed-loop detection system.

Through ADS simulation, it was found that when *R*_b_ increased, it had little effect on the *Q* value. Therefore, when designing the resonator, the substrate coupling capacitance *C*_b_ should be reduced by increasing the thickness of the insulation layer of the substrate, all the electrical routes should be coaxial cable, and the circuit shielding should be performed to reduce the parallel parasitic capacitance *C*_p_.

## 4. Conclusions

In conclusion, a ZnO NW resonator was designed in this study that has a high resonant frequency with real-time closed-loop detection ability and is reliable and highly precise. The parasitic parameters of the resonator were obtained by means of parameter identification, and the influences of these parasitic parameters on the *Q* value were analyzed. Through the experiment and simulation analysis, the following conclusions can be drawn: (i) The top–down processing technology of the NW resonator designed in this paper can protect the NW well, which is beneficial to reducing the noise caused by the defects of the NW itself, and it was found that the phase noise of the resonator is small. (ii) The closed-loop measurement and control system has the ability to detect and send feedback on the weak signal generated by the NW resonator. When the natural frequency of the resonator was locked in a closed loop, the frequency fluctuation value *f*_RMS_ was about 300 Hz, and the FM range could reach 200 kHz. Additionally, the measurement of the resonant frequency of the closed-loop system was directly used to detect the signal generated by the vibration of the resonant beam, which can avoid the interference of some parasitic parameters. (iii) Compared with other parasitic parameters, the parallel parasitic capacitance generated by the device during the test had the greatest influence on the *Q* value. The closed-loop detection system described in this paper can reduce the influence of parasitic parameters on the test and achieve precise closed-loop control of resonators.

## Figures and Tables

**Table 1:** Resonator parameters.

*d* (nm)	*L* (μm)	*ρ* (kg/m^3^)	*U*_0_ (V)	*D* (nm)	*f*_0_ (MHz)	*Q*
70	6.1	5.67 × 10^3^	2	100	10.352	600

**Table 2:** Solution results of equivalent circuit model parameters.

*K* (N/m)	*m* (kg)	*η*	*R_m_* (MΩ)	*C_m_* (fF)	*L_m_* (H)	*C*_0_ (F)
0.289	6.84 × 10^−17^	7.67 × 10^−10^	12.60	2.03 × 10^−3^	116.26	3.40 × 10^−28^
